# Supplementary material for: Black phosphorus ink formulation for inkjet printing of optoelectronics and photonics
Source: Nat Commun. 2017 Aug 17;8:278. doi: 10.1038/s41467-017-00358-1 (PMC5561124; doi:10.1038/s41467-017-00358-1)
Supplement: Supplementary file 1 — Supplementary Information [file 41467_2017_358_MOESM1_ESM.pdf]

File name: Supplementary Information
Description: Supplementary Figures, Supplementary Tables, Supplementary Notes and Supplementary References

File name: Supplementary Movie 1
Description: Inkjet printing of black phosphorus onto 1.5 µm thick polyethylene terephthalate over a 100 mm × 63 mm area. The substrate is laminated onto a photo-paper for ease of handling.

File name: Peer Review File
Description:

Supplementary Note 1. Characterisation of black phosphorus

Supplementary Fig. 1(a) presents the optical absorbance spectra of the solvents (N-Methyl-2-pyrrolidone (NMP), N-Cyclohexyl-2-pyrrolidone (CHP) and isopropanol (IPA)) used for black phosphorus (BP) exfoliation. As observed, there are water signals with all the three solvents in the near infrared wavelength region,[1,2] *i.e.* the peaks at ∼0.91 µm, ∼1.01 µm, ∼1.19 µm and ∼1.39 µm. These signals demonstrate that there is moisture in these solvents. We note that the solvents used in our experiments were purchased in anhydrous composition, with the aim of minimising moisture content in the solutions. Assuming the quality of the chemicals bought from the supplier is as quoted, we suggest that the measured water signals indicate that ambient moisture may have been introduced into the solvents during the handling for absorbance measurement. Indeed, given the solvents are hygroscopic, ambient humidity absorption is inevitable to some degree, and may have contributed to the ∼1% increase in oxidation between bulk and exfoliated BP.

Supplementary Fig. 1(b) presents the optical absorbance spectra of the produced NMP, CHP and IPA based BP dispersions. Subtracting the extinction data shown in Fig. 1(b) with the absorbance data allows us to obtain the dispersion scattering information presented in Fig. 1(c). This absorbance spectra, however, fails to show very clear peaks at ∼465 nm on the log-log scale. We therefore normalise it to 340 nm and re-plot it on linear scale; Supplementary Fig. 1(c). As observed, the NMP spectrum shows only a small peak and that for the CHP only slightly larger, whereas a very prominent peak can be observed in the IPA spectrum. Hanlon *et al.* have shown that this absorbance peak is flake-size dependent, and that it is more prominent for larger flakes.[3] This therefore suggests that the BP flake size distributions in our dispersions are NMP < CHP < IPA, which can be confirmed by the AFM statistics of flake lateral dimension and thickness (Supplementary Fig. 2(d-i)).

As discussed in the manuscript, the NMP, CHP and IPA based BP dispersions exhibit Mie scattering in the wavelength range >500 nm. Supplementary Fig. 2(a-c) show the scattering spectrum (log-log scale) and associated fitting for the three dispersions, where λ is the wavelength and 0.5, 1.5 and 1.9 are the scattering exponents for IPA, CHP and NMP, respectively. The scattering of the three dispersions is linearly fitted to Mie scattering within ∼500-900 nm for NMP, ∼550-1100 nm for CHP, and ∼600-1300 nm for IPA. Treating the BP flakes as non-spherical particles with a characteristic dimensional length l, the relationship between scattering and wavelength can be expressed as a dimensionless size parameter k, where $k = 2\pi l / \lambda$.[4,5] For Mie scattering, $k \sim 1$.[4,5] Therefore, we estimate that the characteristic dimensional lengths of the BP flakes in the three dispersions are approximately 80-145 nm, 90-175 nm and 95-210 nm.

We correlate the estimated characteristic lengths with the flake lateral dimensions measured *via* atomic force microscopy (AFM). To prepare the samples for AFM characterisation, diluted dispersions (5 *vol.%*) are dropcast onto Si/SiO_2. The samples are then dried (initially via nitrogen gun, and subsequently in a vacuum desiccator). The samples are imaged with a Bruker Dimension Icon AFM in ScanAsyst™ mode, using a silicon cantilever with a silicon nitride tip. Supplementary Fig. 2(d-f) present the distributions of BP flake lateral dimensions. The average lateral dimensions correspond well with the characteristic lengths estimated above.

In addition, the scattering exponents, 0.5, 1.5 and 1.9 indicate that BP flake sizes (thickness and lateral dimension) are the smallest in NMP, larger in CHP, and the largest in IPA. The thickness distributions of the three BP dispersions (Supplementary Fig. 2(g-i)) confirm that the average BP flake thickness follows this trend: NMP < CHP < IPA. As demonstrated in Supplementary Fig. 2(d-f), the average lateral dimension also follows the same trend. The flake sizes are thus well correlated with what is expected from the scattering exponents.

Supplementary Fig. 2(j) presents representative AFM images for individual thin BP flakes. As observed, all these flakes show clean surfaces with clearly defined edges, indicating that the flakes in the ink are not oxidised. The lateral dimension of these BP flakes is varied between 70 nm and 400 nm, while the thickness is typically 4-10 nm; Supplementary Fig. 2(k). We note that this size is relatively large compared to the statistics presented in Supplementary Fig. 2(d, g). This is because the AFM measurements are taken under ambient conditions and as such, slow, high resolution imaging of individual, smaller, thinner flakes is challenging due to their increased rate of oxidation (we note here that faster scans, of lower, but sufficient, resolution, are used for the aforementioned gathering of size and thickness distributions). Indeed, we observe that in Supplementary Fig. 2(j) the larger, thicker flakes have sharper edges and more distinct morphologies than the smaller flakes, owing to the size and thickness-dependent rate of degradation over the duration of the scan.

Supplementary Note 2. Raman characterisation

We conduct polarised Raman measurement to check whether Raman spectrum and $I(A_g^1)/I(A_g^2)$ are polarisation independent or not for our solution processed BP and printed BP samples. The sample for this polarised Raman measurement is prepared by dropcasting the BP ink onto a Si/SiO_2, subsequently dried under nitrogen. Polarised Raman characterisation of the BP sample is taken at one single point, using an excitation wavelength of 514 nm with a power <0.1 mW and a duration of 10 s for each polarised angle. Supplementary Fig. 3(a) presents the polarisation-resolved Raman spectra. The peak intensities here are normalised to $I(A_g^2)$. As shown, the spectra do not show any observable variations under the varied polarisation angles,

suggesting that Raman spectra are independent on polarisation. The relationship between the peaks, A_g^1 and A_g^2, has been used as an indiction for BP oxidation.[3,6] The acquired peak intensity ratio, $I(A_g^1)/I(A_g^2)$ remains constant (0.480 ± 0.04) under the varied polarisation angles (Supplementary Fig. 3(b)), demonstrating that $I(A_g^1)/I(A_g^2)$ is also polarisation independent. We suggest the reason accounting for this polarisation independence here is that we are studying dropcast and printed BP flakes and as such, there is no alignment in the orientation of the deposited BP flakes. During measurement, within the area of the laser spot (∼1 μm) there are many flakes distributed in random orientation to one another. As we have demonstrated, this seems to nullify any polarisation dependence that is otherwise observed in literature.[7–9]

Preceding studies show the full-width at half maximum (FWHM) are all within ∼2-6.5 cm^{-1} (A_g^1), ∼2-7 cm^{-1} (B_{2g}), and ∼2-8 cm^{-1} (A_g^2) for 1-6 layer of mechanically exfoliated and bulk BP.[6] In Supplementary Fig. 4(a) (*i.e.* Fig. 1(d) with FWHM labels), we show that the typical FWHM of our exfoliated and bulk BP samples are consistent with those in literature. We further acquire the FWHM statistics from the Raman mapping measurements (∼360 measurement points) of exfoliated and bulk BP; Supplementary Fig. 4(b). We demonstrate that the FWHM statistics are also consistent with those reported in literature. The consistency in the FWHM suggests high crystallinity of our exfoliated BP.

The Raman peaks can be susceptible to the experimental conditions, and as such the intensity may be below the noise or sensitivity level of the spectrometer. Indeed, as indicated by the grey areas in the Raman mapping (consisting of 20 × 20 data points) - Supplementary Fig. 5(a) (see Fig. 1(e)), we find that the Raman intensity of 10% of the data points is not strong enough for interpretation. We plot a representative Raman spectrum of this type as #1, Supplementary Fig. 5(b). These low intensity data points are discarded for BP oxidation investigation. We also show a representative Raman spectrum with $I(A_g^1)/I(A_g^2)$ within the threshold 0.2-0.6 as #2, and outside as #3, respectively; Supplementary Fig. 5(b). These remaining (∼360) data points have strong Raman intensity for BP oxidation investigation. In conclusion, we feel that our protocol is sufficiently reliable for oxidation investigation.

Here we note that though a ratio value of 0.2-0.6 is used as the threshold for BP low oxidation by Favron *et al.*,[6] there are other studies (for instance, Hanlon *et al.*[3]) which consider >0.6 for low oxidation and <0.6 for high oxidation. We have independently analysed the $I(A_g^1)/I(A_g^2)$ Raman mapping histograms for freshly cleavaged bulk BP (Supplementary Fig. 13(a)), BP NMP dispersion (Fig. 1(f)), and printed BP (Fig. 4(d)). These BP samples represent three different oxidation status. For the freshly cleavaged bulk BP sample, in which case high oxidation is very unlikely, the majority of ratio values are observed within 0.2-0.6, with only 3.1% >0.6. For the NMP dispersion and printed BP that with higher possibilities of oxidation due to the exfoliation, ink formulation and printing processes, the percentage for >0.6 increases up to 4.18% and 10.03%, respectively. This, in conjunction with the above references, suggests that >0.6 indicates a high oxidation, and 0.2-0.6 a low oxidation. We thus use 0.2-0.6 for minimal oxidation.

We note Hanlon *et al.* used peak intensity ratio $I(A_g^1)/I(A_g^2)$,[3] while Favron *et al.* integrated peak intensity ratio.[6] We have also conducted our independent experiments to shed light on this; Supplementary Table 1. We show here that the Raman mapping of $I(A_g^1)/I(A_g^2)$ is consistent with BP oxidation process, suggesting that it is a reasonable tool to work as an indication for oxidation of our solution processed and printed BP. The integrated peak intensity ratio, however, is not consistent. It even indicates a high oxidation for freshly cleavage bulk BP, in which case significant oxidation is unlikely. Therefore, we then use the peak intensity ratio, $I(A_g^1)/I(A_g^2)$ to investigate BP oxidation.

	intensity ratio outside 0.2-0.6 (%)	integrated intensity ratio outside 0.2-0.6 (%)
Bulk BP	3.1	16.1
Bulk BP with parylene-C	5.8	23.1
Dropcast BP NMP dispersion	4.2	1.7
Printed BP	10.0	90.0
Printed BP with parylene-C	23.3	18.0

Supplementary Table 1. Raman intensity ratio and integrated intensity ratio outside 0.2-0.6

Supplementary Note 3. Ink formulation and stability

IPA is used as the major ink carrier solvent for ink formulation in our work. Though we acknowledge that IPA does have a level of toxicity, it is comparably benign when compared to the solvents widely used in the literature for inkjet ink formulations containing 2d materials, including graphene and transition metal dichalcogenides, which are typically based on harsh, toxic solvents such as N-Methyl-2-pyrrolidone (NMP).[10,11] Such organic solvents are not only demonstrably harmful for human health and the environment, but can also be incompatible with many polymeric substrates, limiting the applications of these inks. It is, in part, specifically to avoid using these toxic solvents, that we are formulating the ink using IPA. Quoting the

safety data sheet provided by Sigma Aldrich, the acute toxicity limits for IPA are oral - 5,045 mg per kg (NMP: 3,914 mg per kg), inhalation - 16000 ppm (NMP: 5100 ppm) and dermal - 12,800 mg per kg (NMP: 8,000 mg per kg), and the UK workplace exposure limit is 500 ppm (NMP: 10 ppm). Given its relatively low hazardous potential, IPA is widely used in various commercial functional and pigment-based inks, *e.g.* commercial silver nanoparticle inkjet inks. IPA will also not cause damage to commonly used polymeric substrates, making it compatible for the development of flexible, printed devices. In addition, as we have demonstrated in the manuscript, IPA is possible to formulate an ink for high loading ($\sim$5 gL^{-1}), stable single-droplet jetting, and appropriate wetting of the substrates. The low boiling point of IPA (82.6 °C) can also allow a rapid ink drying ($<$10 s) at low temperatures ($<$60°C). This rapid ink drying, in combination with the high ink loading, leads to a significantly reduced printing time, giving low possibilities for BP oxidation. This is of vital importance for inkjet printing of BP.

We have investigated our ink formulation with varied 2-butanol volume percentages. Here we attach optical micrographs for dried droplets formulated with 0 *vol.%*, 10 *vol.%*, and 20 *vol.%* 2-butanol; Supplementary Fig. 6(a). 0 *vol.%* is $IPA_{S.E.}$, and 10 *vol.%* is the ink in the manuscript; see Fig. 2(f). The droplets are all $\sim$10 pL and inkjet-printed onto untreated Si/SiO_2 and dried at 60°C. As we can observe, 0 *vol.%* forms a noticeable coffee ring effect, while both 10 *vol.%* and 20 *vol.%* do not. The lack of coffee ring suggests that a surface tension gradient is generated to induce Marangoni flow within the droplets in both these cases. We further study the time-dependant contact angle of the three formulations; Supplementary Fig. 6(b). Contact angle for 0 *vol.%* and 10 *vol.%* are quoted from Fig. 2(c) in the manuscript. The absence of large variations for 10 *vol.%* and 20 *vol.%* confirms the lack of coffee ring effect in these two cases. However, the droplet diameter increases as the volume percentage of 2-butanol increases, with $\sim$75 µm for 10 *vol.%* and $\sim$85 µm for 20 *vol.%*. As shown in Supplementary Fig. 6(b), the contact angle of 20 *vol.%* decreases faster than that of 10 *vol.%* during the drying process, suggesting that 20 *vol.%* spreads faster and in a larger area than 10 *vol.%*. This explains the lager dried diameter of 20 *vol.%*. An increase in drop diameter is undesirable as it means a decrease in printing resolution. Based on the above considerations on the coffee-ring effect and the printing resolution, we chose 10 *vol.%* for the ink formulation.

Meanwhile, in Fig. 2(f,g) we show that BP-$IPA_{S.E.}$ delivers a more even distribution of flakes than the NMP dispersion. We propose such alleviation of the coffee ring effect is due to the combination of better wetting (*i.e.* lower contact angle $<$30°) and faster drying time of BP-$IPA_{S.E.}$ ($\sim$30 s) when compared to the NMP dispersion ($\sim$60°, $\sim$12 hours) under the same measurement conditions (2 µL droplet, $\sim$20°C). Therefore, for the ink formulation, the combination of the recirculating Marangoni flow and the rapid drying ensures minimisation of the coffee ring effect for uniform material deposition.

After ink formulation, we also employ UV-Vis optical extinction spectrum to verify the concentration of the final ink; Supplementary Fig. 7(a). The ink is diluted to 1 *vol.%* for the measurement to avoid absorbance saturation. Since the extinction value at 660 nm is 0.133, we verify the ink concentration as $\sim$5 gL^{-1} using the extinction coefficient, 267 $Lg^{-1}m^{-1}$ at 660 nm.[12]

After ink formulation, we again investigate the scattering spectrum of the produced ink to assess whether there are any possible flake aggregations associated with the solvent transfer process. Supplementary Fig. 7(b) presents the scattering spectra and associated scattering fitting for the ink and the BP NMP dispersion. The ink is diluted to 1 *vol.%* to avoid absorption saturation, and 1 *vol.%* is used here also to keep the diluted ink concentration consistent with that of the BP NMP dispersion, which is 10 *vol.%* diluted for measurement. The normalised scattering of the ink shows a difference compared to that of the NMP dispersion. However, both the spectra can be fitted with a scattering exponent of $\sim$1.9. This suggests that the BP flakes in the ink do not have large variations in flakes sizes, and therefore that the ink formulation procedures do not cause aggregation of the BP flakes.

As demonstrated, the ink carrier (IPA/2-butanol) affords the production of a highly-concentrated BP ink. However, before conducting printing processes, it is necessary to assess the stability of ink against sedimentation. We develop a homemade stability measurement system to address this. We employ a 632 nm laser beam through the ink (diluted to 5 *vol.%* to avoid absorption saturation), and collect the laser intensity transmitted through the diluted ink over one week with 5 mins interval. The laser intensity transmitted through the ink carrier, IPA/2-butanol, is also collected as the base laser intensity. The acquired light intensity absorbed by the BP flakes, *i.e.* the difference between the base laser intensity and the laser intensity transmitted through the diluted ink, is plotted as normalised absorption in Supplementary Fig. 8(a). The absorbed intensity shows only a 1% drop over 180 hours, indicating $<$1% BP flakes sediment. This demonstrates the high stability of the ink against sedimentation over a timeframe that would prove viable for large-scale ink production and printing.

We now assess the stability of ink itself against oxidation. We have conducted Raman $I(A_g^1)/I(A_g^2)$ ratio mapping of a dried dropcast ink sample using a formulation prepared two months previously and stored under nitrogen in the interim. Here we attach the acquired intensity ratio histogram; Supplementary Fig. 8(b). It shows that the proportion outside 0.2-0.6 has increased to 21.69%. Therefore, whilst this is not an excessive increase given the timescale, it is clear that it is best to use freshly prepared BP ink for device fabrications.

We propose that this oxidation of the ink may arise from the moisture and oxygen trapped in the ink carrier. The ink carrier

solvents, *i.e.* IPA and 2-butanol, used were purchased in anhydrous composition, with the aim of minimising moisture content. However, the measured optical absorbance (Supplementary Fig. 8(c)) of the ink carrier also shows notable water signal peaks at ∼0.91 µm, ∼1.01 µm, ∼1.19 µm and ∼1.39 µm. This suggests that there was moisture introduced into the ink carrier during the handling and formulation processes. We believe that this moisture in addition to trapped oxygen contributes to the degradation to BP in the stored ink. If it is indeed the case, we argue that it is possible to have long shelf life with our BP ink as long as the ink formulation and subsequent storage takes place in a controlled atmosphere.

As demonstrated above, the IPA/2-butanol mixture affords a BP ink stable against sedimentation over a timescale of weeks. An accepted guideline for stable inkjet printing is that the average particle size should be $<1/50^{th}$ of the nozzle diameter (22 µm).[10] The AFM measurements of our flakes indicate that they are ∼80 nm in lateral size (Supplementary Fig. 2(d)), significantly smaller than this threshold. The combination of the ink stability against sedimentation and the small size nature of the flakes therefore allows stable, long, large-scale printing processes. We have uploaded a supplementary video taken during a long printing session (over 6 hours) on a printing scale of 100 mm × 63 mm. To prevent build-up of BP flakes on the nozzles (which could ultimately lead to clogging) across different printing sessions, we conduct 2-3 cleaning cycles of the nozzles *via* purging the nozzles with the IPA/2-butanol mixture before and after each printing session, using the printer's built-in cleaning cycles.

Supplementary Note 4. Characterisation of printing morphology

As shown in Fig. 3(b) in the manuscript, the edge roughness of the printed lines is a key characteristic of the printing morphology. Here, we present the measurement scheme of the line edge roughness. The edge roughness is defined as $(L_x - L_y) / 2$, where L_x and L_y are the widths of a printed line at its widest and narrowest points, respectively. To distinguish deviations caused by excess ink (as in the case of stacked coins and bulging) from those caused by insufficient ink (as in the case of scalloping and individual droplets), we set the roughness of stacked coins and bulging as negative, and scalloping and individual droplets as positive. For example, Supplementary Fig. 9(a), showing a line printed with 25 µm droplet spacing at 60°C, exhibits a maximal width L_1 ∼130 µm, and a minimal width L_2 ∼115 µm. The roughness therefore can be calculated as $(L_1 - L_2) / 2$ = (130 - 115) / 2 µm = 7.5 µm. However, since this line is broadened by excess ink and forms bulging, we set the calculated edge roughness value as negative, *i.e.* -7.5 µm. For the case presented in Supplementary Fig. 9(b), which is a scalloped line printed with a droplet spacing of 85 µm at 60°C, we set its roughness as positive. Therefore, the roughness is calculated as $(L_3 - L_4) / 2$ = (71 -32) / 2 µm = 19.5 µm.

We measure the diameter of isolated droplets printed at different heating temperature. The relationship between the diameter and the temperature is presented in Supplementary Fig. 9(c). As shown, the droplet diameter is inversely related to the temperature.

We show in Fig. 4(a) that the optical extinction for 1 and 2 printing repetitions has a relatively large divergence from the linear fitting though the overall variation is <2% between 1-10 printing repetitions. As presented in Supplementary Fig. 9(d), the optical extinction is divergent by ∼110% from the fitted extinction for 1 printing repetition and ∼30% for 2 printing repetitions. For 3 printing repetitions, it drops to ∼5% whilst it is only ∼1% for 10 printing repetitions.

Supplementary Note 5. Characterisation of optical properties of printed BP

The spatial homogeneity of printed BP is characterised by raster-scanning printed BP patterns through an open-aperture Z-scan set-up, which allows the intensity dependent transmission to be recorded as a function of position on the sample. The Z-scan set-up utilises an erbium fibre laser operating at 1562 nm, with 150 fs pulse duration at a repetition frequency of 10 MHz. Supplementary Fig. 10 shows a typical optical absorption profile of printed BP on PET obtained from a Z-scan experiment. The data can be fitted using a simple two-level saturation model: $\alpha(I) = (\alpha_l - \alpha_{ns}) / (1 + I / I_{sat}) + \alpha_{ns}$,[13,14] where α_l is the linear absorption at low intensity and α_{ns} is the nonsaturable absorption at high intensity, I is the instantaneous intensity, and I_{sat} is the saturation intensity. The modulation depth (α_d) of a device is given by: $\alpha_d = \alpha_l - \alpha_{ns}$. Consequently, I_{sat} can be defined as the intensity required to reduce the absorption $\alpha(I)$ to $\alpha_l - (\alpha_d / 2)$. Therefore, from the fit we can acquire α_l, α_{ns} and I_{sat} as 8.71%, 5.05% and 7.5 $MWcm^{-2}$ respectively.

Supplementary Note 6. Stability of printed BP against oxidation

In the manuscript, we have demonstrated the stability of the encapsulated printed BP using the optical extinction of the sample at 550 nm. Here, we compare the extinction spectrum of the as printed and encapsulated printed BP samples across the 350-850 nm range; Supplementary Fig. 11. The extinction spectrum continuously decreases during the measurement period for the as printed BP sample. This trend continues even at the end of this period, in line with what we observe in Fig. 4(e). For the encapsulated printed BP sample, the extinction spectrum shows a small decrease (<5%) during the first 5 days. However, it then

stabilises and exhibits no noticeable change across the 350-850 nm wavelength range for the remainder of the measurement period. This further confirms that the encapsulated printed BP is well protected by parylene-C.

We carry out further investigations on BP stability using Raman spectroscopy. Raman map (Fig. 4(c)) and associated histogram (Fig. 4(d)) shows the intensity ratio, $I(A_g^1)/I(A_g^2)$ of the printed BP samples. Figure 4(d) is reproduced as the Supplementary Fig. 12(a) for clarity. The histogram suggests a ∼10% oxidation proportion after printing. We then study this intensity ratio, $I(A_g^1)/I(A_g^2)$ of the printed samples immediately after encapsulation with parylene-C and the same sample on the 13th and the 30th day; Supplementary Fig. 12(b-d). The corresponding ratio values outside the 0.2-0.6 range is 23.3%, 34.3% and 33.9%, respectively. Thus, the $I(A_g^1)/I(A_g^2)$ value shows a large increase immediately after encapsulation. This then increases to ∼33% on 13th day and stabilises (30th day).

To investigate the large increase in the $I(A_g^1)/I(A_g^2)$ ratio, we prepare a set of freshly cleaved bulk BP sample with and without the parylene-C encapsulation. The corresponding ratio values outside the 0.2-0.6 is 3.1% and 5.8%, respectively; Supplementary Fig. 13. We additionally note that the average value of $I(A_g^1)/I(A_g^2)$ for both the freshly cleaved bulk BP and the printed BP samples increase after encapsulation; Supplementary Fig. 12 and Supplementary Fig. 13. As shown by Favron *et al.*, $I(A_g^1)$ can be easily affected by the perturbations from contacting substances, unlike $I(A_g^2)$.[6] We therefore propose that the large increase in $I(A_g^1)/I(A_g^2)$ after encapsulation can be partially attributed to the increase in $I(A_g^1)$ due to close contact with parylene-C. As noted in the manuscript, the larger surface area of the printed BP compared to bulk BP lead to a larger contact area with parylene-C and hence, larger change in the $I(A_g^1)/I(A_g^2)$ value. The increase from 23.3% (Supplementary Fig. 12(b)) to ∼33% (Supplementary Fig. 12(c, d)) is likely due to limited oxidation of trapped oxygen and moisture. We therefore conclude that the absolute value of the ratio $I(A_g^1)/I(A_g^2)$ may not truly represent the oxidation proportion of BP samples coated with parylene-C.

Supplementary Note 7. Characterisation of mode-locked ultrafast laser

The configuration of the erbium-doped ultrafast fibre laser set-up is shown in Supplementary Fig. 14(a). This set-up consists of single-mode all-fibre integrated components for alignment-free and compact system. The fibre amplifier is composed of a 0.7 cm long single-mode erbium-doped active fibre (LIEKKI Er-8/125), which is co-pumped by a 980 nm pump laser diode. In addition, this set-up includes a polarisation-independent optical isolator to ensure unidirectional propagation, a 10:90 fused fibre output coupler for both spectral and temporal diagnostics, and a polarisation controller to enable a thorough and continuous adjustment of the net cavity birefringence, but that is not fundamental to the mode-locking action.

Mode-locking of the ultrafast laser set-up is operated at 1562 nm, with intra-cavity intensities reaching 32.7 $MWcm^{-2}$. Self-starting mode-locking is achieved at the fundamental repetition frequency of 31.6 MHz. To evaluate the operation stability of the printed BP-SA and the mode-locking performance of the fibre laser, we record the parameters of the output pulses every 6 hours for over 30 days. In addition to the stable performance presented in Fig. 5(b, c, d), Supplementary Fig. 14(b) presents the radio frequency (RF) spectrum for same period, also showing no noticeable variations. This demonstrates an excellent long-term mode-locking stability.[15] The output pulse duration, measured using an intensity autocorrelator, is 605 fs (deconvolved), well fitted with a $sech^2$ pulse shape; Supplementary Fig. 14(c).

Supplementary Table 2 presents the reported results in literature of BP SAs fabricated both through solution-processing based techniques and otherwise as a comparison to our work. The SAs show excellent device operation under intense irradiation for over 714 hours, at least 24 times longer than those previously reported.

Supplementary Note 8. Fabrication and characterisation of graphene/Si Schottky junction photodetector

The BP/graphene/Si Schottky junction photodetector (BP/Gr/Si) is fabricated by inkjet printing the BP ink onto the Si window of a graphene/Si Schottky junction photodetector (Gr/Si), followed by encapsulation of 100 nm thick parylene-C. Supplementary Fig. 15(a) schematically illustrates the fabrication process of Gr/Si, and Supplementary Fig. 15(b, c) the height profile and top-view of Gr/Si, respectively.

The fabrication process of Gr/Si is as follows: (1) A Si/SiO_2 wafer (SiO_2 thickness 100 nm) is cleaned with acetone/IPA/DI water. (2) The Si/SiO_2 wafer is first patterned through e-beam lithography. Subsequently a 50 nm gold (Au) layer is deposited, followed by lift off. This forms the Au electrode pattern, enclosing a Si/SiO_2 window (∼460 μm × 460 μm). (3) The device is further patterned to give a SiO_2 window of ∼450 μm × 450 μm, followed by etching with hydrofluoric acid. The exposed SiO_2 is etched away to give a Si window ∼450 μm × 450 μm. (4) Monolayer graphene grown on copper (Cu) foil by chemical vapour deposition (CVD) is then transferred onto the device, covering the Au electrode and the Si window.

The growth of the CVD graphene uses an Aixtron Black Magic CVD system. Prior to CVD growth, 25 μm thick Cu foil is pre-treated in 5% Nitric acid for 5 s, followed by multiple rinsing in DI water, to remove Cu imperfections and native oxide layer. The Cu foil is then loaded into the chamber of the CVD system and annealed at 1060°C for 1 hour under Hydrogen

Fabrication method	α_d (%)	Laser type	Laser properties		Demonstrated stability (hours)	Reference
			λ (nm)	τ		
Inkjet printing	8.7	Er fibre	1562	605 fs	>714	Our work
ME	8.1	Er fibre	1571	946 fs	28	16
ME	7.5	Er fibre	1561	272 fs	-	17
ME	0.6-4.6	Tm fibre	1910	739 fs	-	18
ME	-	Er fibre	1559	786 fs	-	19
ME	9.8	Er fibre	2783	42 ps	-	20
ME	8	Yb fibre	1086	7.54 ps	-	21
ME	6.9	Er fibre	1561	2.66 ps	-	22
ME	-	Er fibre	1559	805 fs	-	23
LPE	21	Er fibre	1560	670 fs	-	24
LPE	0.8	Er fibre	1561	1.44 ps	-	25
LPE	41.2	Ho/Pr fibre	2867	8.6 ps	-	26
LPE	6.91	Er fibre	1532-1570	940 fs	10	27
LPE	4.1	Er fibre	1558	2.18 ps	-	28
LPE	50-90	Tm/Ho fibre	1880-1940	1.58 ps	2	29
LPE	19	Er fibre	1568	117.6 ns	-	30
LPE	10.1	Er fibre	1569	280 fs	24	31

Supplementary Table 2. Mode-locked fibre lasers using BP SAs. ME - mechanical exfoliation; LPE - liquid phase exfoliation; α_d - modulation depth; λ, operating wavelength; τ, pulse duration.

gas (350 sccm) and a constant pressure of ∼10 mBar to obtain a smooth surface and increase the Cu grain size. Next, 2 sccm Methane gas is introduced with 350 sccm Hydrogen gas for 20 min for the growth of graphene. Finally, a quick cooling to room temperature is performed at a rate of 300°C per minute.

We characterise the CVD graphene after it is transferred onto the Si/SiO_2 substrate through Raman spectroscopy; Supplementary Fig. 15(d). The red dot in the Supplementary Fig. 15(c) indicates the position of Raman characterisation. The excitation wavelength is 532 nm, with a power of ∼1 mW. In the Raman spectrum, two main peaks at ∼1589 cm^{-1} (G peak) and ∼2700 cm^{-1} (2D peak) are observed. No noticeable D peak is seen, which is commonly associated with structural defects.[32] The absence of the D peak therefore is a direct indication of high quality for our CVD graphene.

We measure the devices with four different laser output powers for each wavelength in our work: 2.38, 256.8, 513 and 1200 μW for 450 nm, and 5.9, 10.6, 15.28 and 19.9 mW for 1550 nm. The laser beam diameter is ∼2 mm, giving a beam spot area of 3.14 mm^2. The estimated power densities are therefore 0.76, 81.78, 163.38 and 382.17 Wm^{-2} for 450 nm, and 1.88×10^3, 3.38×10^3, 4.87×10^3 and 6.34×10^3 W/m^2 for 1550 nm, respectively. The photoactive region of our photodetector is 450 μm × 450 μm. Therefore, the actual incident laser powers on the photoactive region are 0.15, 16.56, 33.08 and 79.11 μW for 450 nm, and 0.38, 0.68, 0.99 and 1.28 mW for 1550 nm, respectively.

Supplementary Fig. 16(a) presents the dark currents of Gr/Si and BP/Gr/Si in the same figure on log-log scale for a better comparison. As shown, the dark current of Gr/Si when reversed biased is on the ∼1 nA scale, while it is ∼100 nA for BP/Gr/Si. This could arise from the doping of the CVD graphene by the printed BP layer discussed in the manuscript, and may have been responsible for the improvement in the device performance in terms of photocurrent change. Since we are here studying the effect of printed BP on device performance, we therefore do not scale up the photocurrent changes of the Gr/Si to compare those of the BP/Gr/Si.

As we can observe, indeed the forward photocurrent changes are small (up to ∼7.5 μA; Fig. 5(f, g)) compared to the dark currents (up to ∼900 μA). However, the reverse photocurrent changes are on the μA scale (up to ∼12.5 μA; Fig. 5 (f, g)), significantly different from the dark currents (on the nA scale, up to ∼100 nA). This demonstrates the printed BP layer does indeed lead to a device performance improvement. In showing this, we aim to discuss the potential of our BP ink for printed optoelectronics and photonics.

Supplementary Fig. 16(b) presents the time response of Gr/Si and BP/Gr/Si at 1550 nm. Gr/Si shows no response, whereas BP/Gr/Si exhibits a stable and reproducible response. The response time of the cycle shown in Fig. 16(c) is ∼0.55 ms, and the recovery time is ∼1.09 ms.

We further assess the operation stability of BP/Gr/Si. Supplementary Fig. 17 presents the photocurrent changes and the time response of BP/Gr/Si at 1550nm after 7 days (168 hours) exposure to ambient conditions. As shown, we find negligible

changes in both the photocurrent changes and the time response. This demonstrates the high operation stability of the device.

Fabrication	Structure	Properties			Demonstrated stability (hours)	Reference
		Spectral range	Responsivity (mAW^{-1})	Response time		
Inkjet printing	Schottky junction	visible - near IR	164	0.55 ms	>168	Our work
ME	FET	visible - near IR	20	-	-	33
ME	FET	visible - near IR	4.8	1 ms	-	34
ME	FET	mid IR	82	-	-	35
ME	FET	terahertz	0.15 (VW^{-1})	-	-	36
ME	Waveguide	near IR	657	-	-	37
ME	p-n junction	visible - near IR	1.5	40 μs	-	38
ME	Heterojunction	visible	418	-	-	39
ME	Heterojunction	visible - near IR	22.3	15 μs	-	40

Supplementary Table 3. Photodetector based on BP. ME - mechanical exfoliation; LPE - liquid phase exfoliation; IR - infrared; FET - field effect transistor

Application of BP in photodetectors have been reported already; Supplementary Table 3. However, these devices are fabricated with mechanically exfoliated BP flakes. This material production technique suffers from extremely low yield and high uncontrollability. Therefore, this device fabrication technique is highly limited by the material production yield, and it requires high device fabrication complexity and cost. To the best of our knowledge, this is the first report of printable BP photodetectors. The produced visible to near-infrared photodetector exhibits high responsivities (up to 164 mAW^{-1}), fast response (up to $\sim$0.55 ms), and a long-term (>7 days) operation stability. Also, this printable technology enables the benefits of high yield, low cost, reduced fabrication complexity, as well as potentially the thin-form factor, flexibility, and stretchability of the fabricated photodetectors.

In addition to the SAs and photodetector we have demonstrated already *via* inkjet printing of BP, we envision that our BP ink formulation also holds huge potential in other applications, such as printable electronics and printable energy storage. Though there are no reports of such BP based printable devices yet, we have seen many successful demonstrations of proof-of-concepts based on printing of other functional material systems. For instance, inkjet printed graphene transistors developed by Torrisi *et al.*,[10] all inkjet printed 2d material based read-only memories developed by McManus *et al.*,[41] and all inkjet printed carbon nanotubes based flexible supercapacitors developed by Choi *et al.*[42] The BP ink can be effortlessly transferred to the fabrication of such devices.

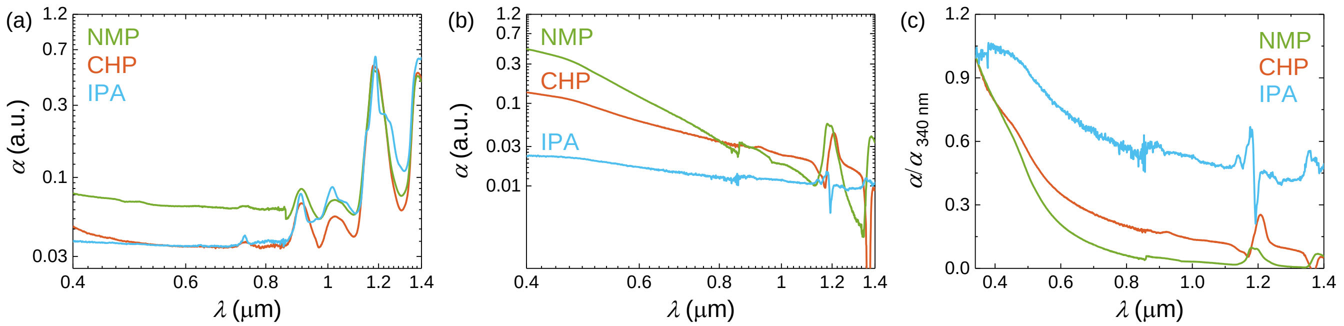

Supplementary Figure 1. (a) Optical absorbance (log-log scale) of the anhydrous NMP, CHP and IPA used for BP exfoliation; Optical absorbance spectra (b) on log-log scale and (c) normalised to 340 nm on linear scale of the produced NMP, CHP and IPA based dispersions.

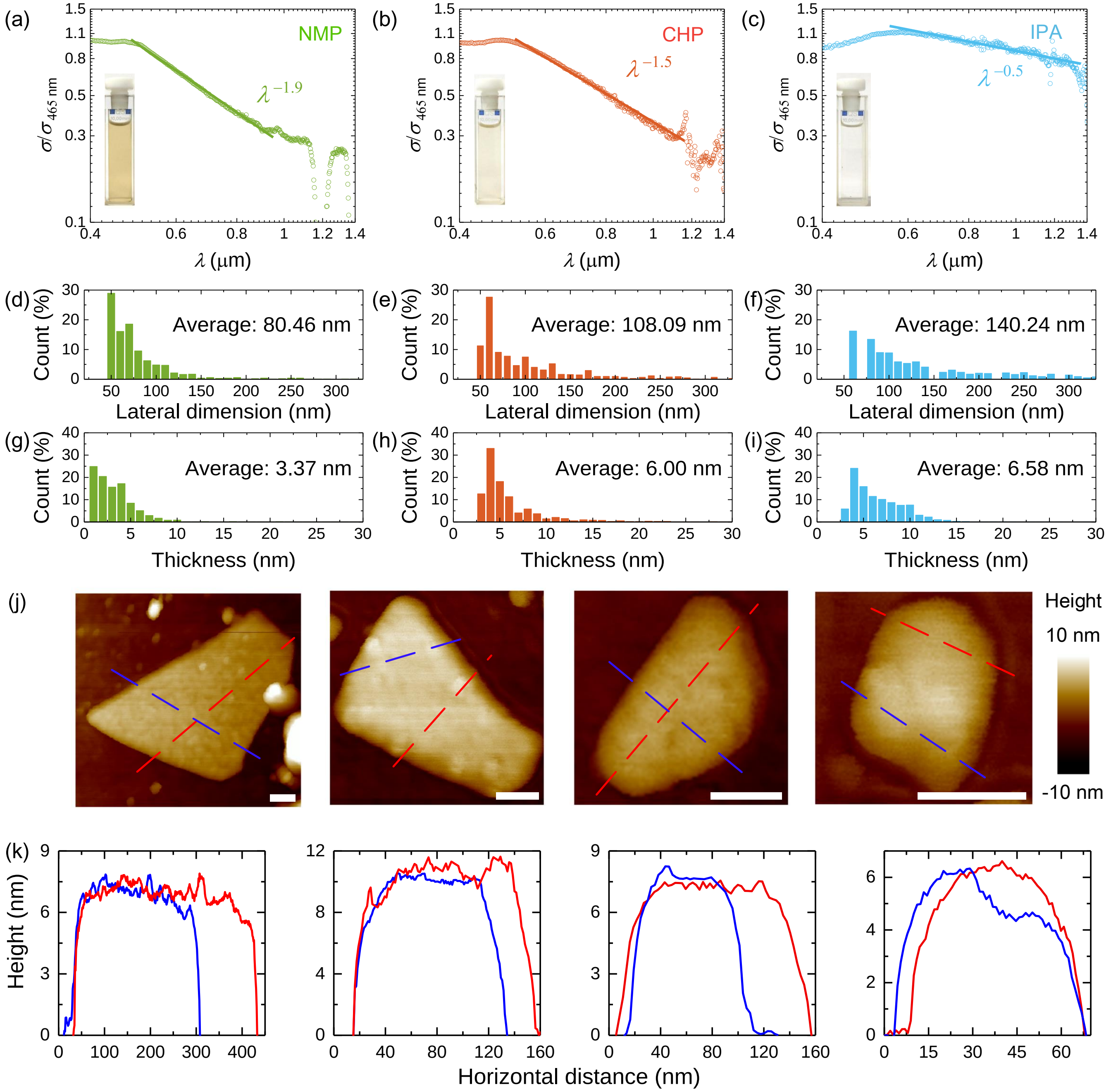

Supplementary Figure 2. Light scattering (a, b, c), and the corresponding distributions in lateral dimension (d,e,f) and flake thickness (g, h, i) measured via AFM for the NMP, CHP and IPA dispersions; (j) Representative AFM images for individual BP flakes and (k) corresponding height profiles, scale bar - 50 nm. The plots in (a, b, c) are reproduced from Fig. 1(c) for ease of comparison, and the insets showing the diluted dispersions (10 *vol.%*).

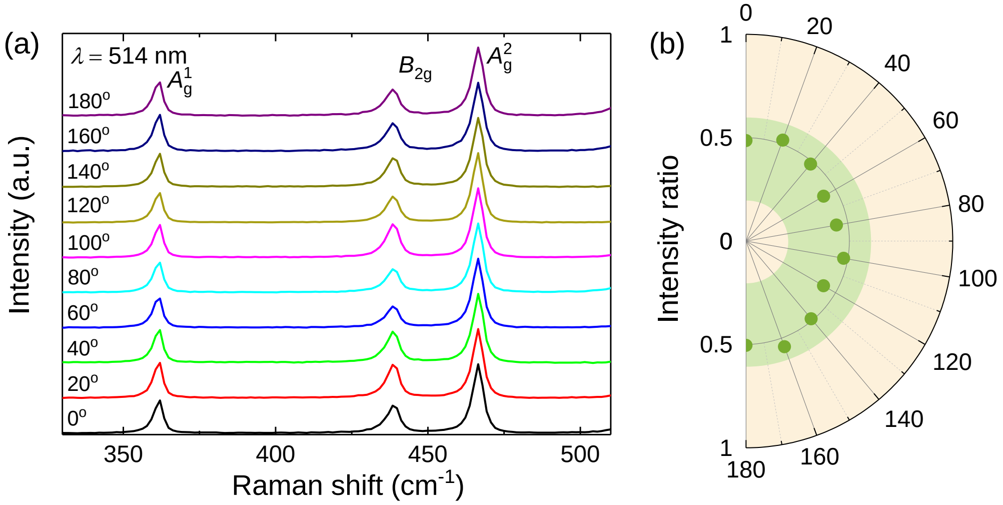

Supplementary Figure 3. (a) Polarisation-resolved Raman spectra of dropcast dried BP ink, intensity normalised to $I(A_g^2)$; (b) The associated peak intensity ratio, $I(A_g^1)/I(A_g^2)$. The green region corresponds to low oxidation, and the yellow region high oxidation.

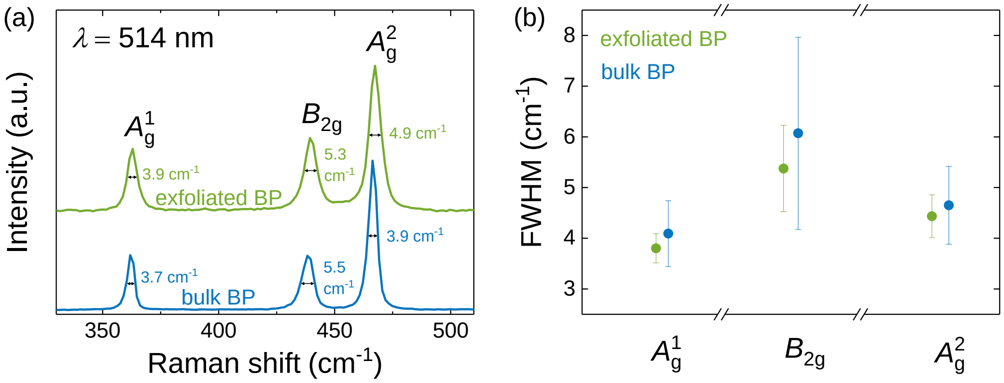

Supplementary Figure 4. (a) Typical Raman spectrum with FWHM for the exfoliated and bulk BP, *i.e.* Fig. 1(d) with FWHM labels; (b) FWHM statistics of ∼360 measurement points.

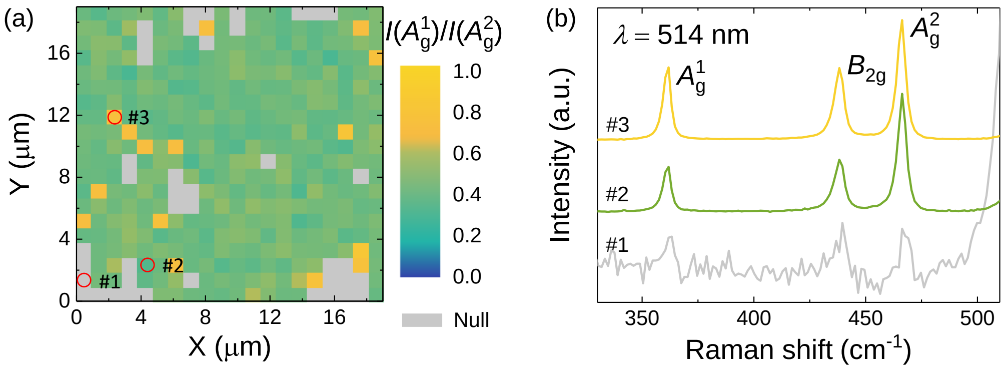

Supplementary Figure 5. (a) Raman map of the intensity ratio, $I(A_g^1)/I(A_g^2)$, with 1 μm spatial step. The grey squares correspond to regions where the Raman intensity is too low for accurate interpretation; (b) Raman spectra of #1, #2 and #3.

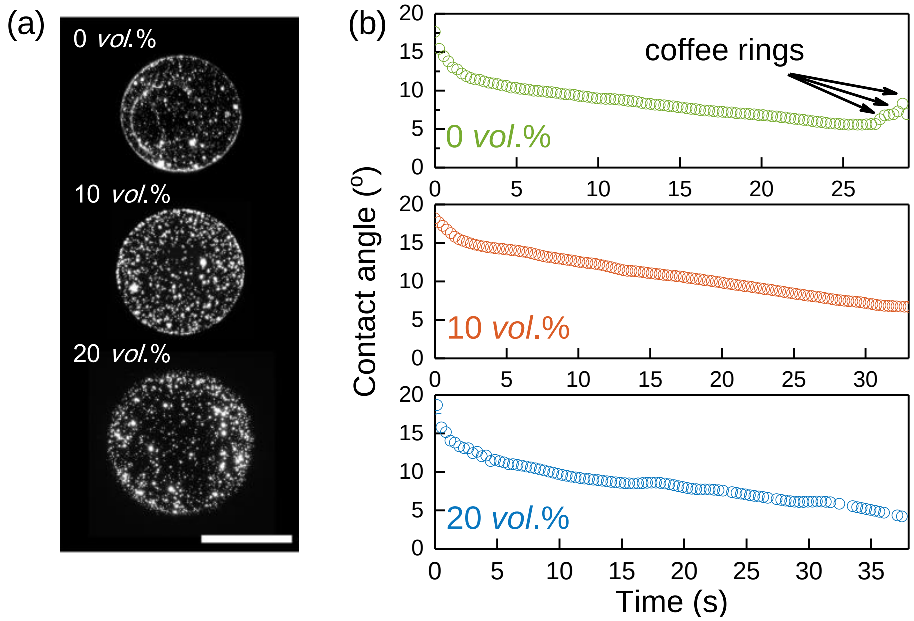

Supplementary Figure 6. (a) Optical micrograph of dried droplets on untreated Si/SiO_2 formulated with 0 *vol.%*, 10 *vol.%* and 20 *vol.%* 2-butanol, scale bar - 50 µm; (b) Change in contact angle during droplet (∼2 µL) drying process on untreated Si/SiO_2 at room temperature.

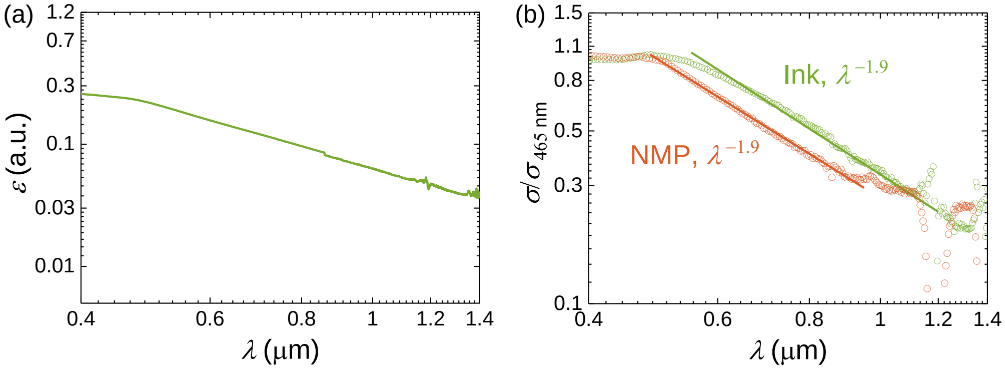

Supplementary Figure 7. (a) Optical extinction (log-log scale) of the ink, the ink is diluted to 1 *vol.%*; (b) Optical scattering with associated fitting (log-log scale) of the BP NMP dispersion and the diluted ink. Scattering is normalised to the 465 nm peak.

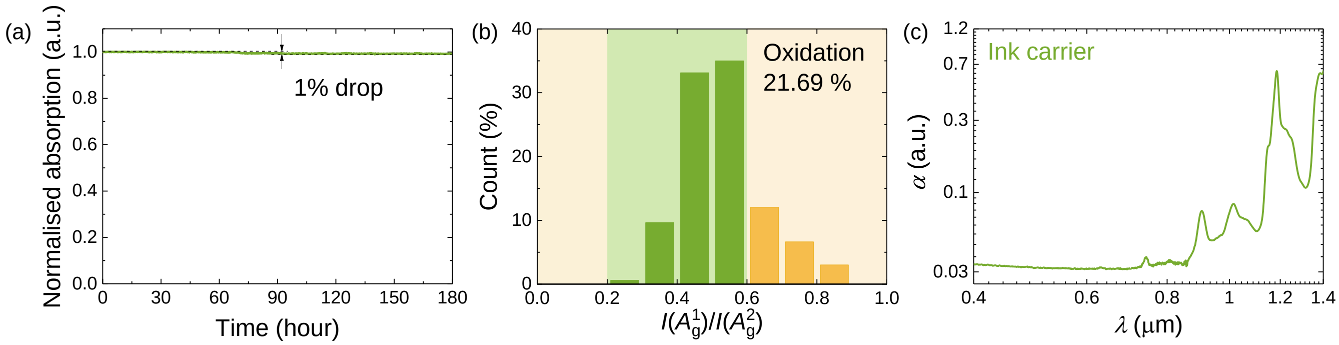

Supplementary Figure 8. (a) Ink absorption against time, the ink is diluted to 5 *vol.%*; (b) $I(A_g^1)/I(A_g^2)$ histogram for dropcast dried BP ink, the ink has been kept under nitrogen for 2 months; (c) Optical absorbance (log-log scale) of the IPA/2-butanol ink carrier.

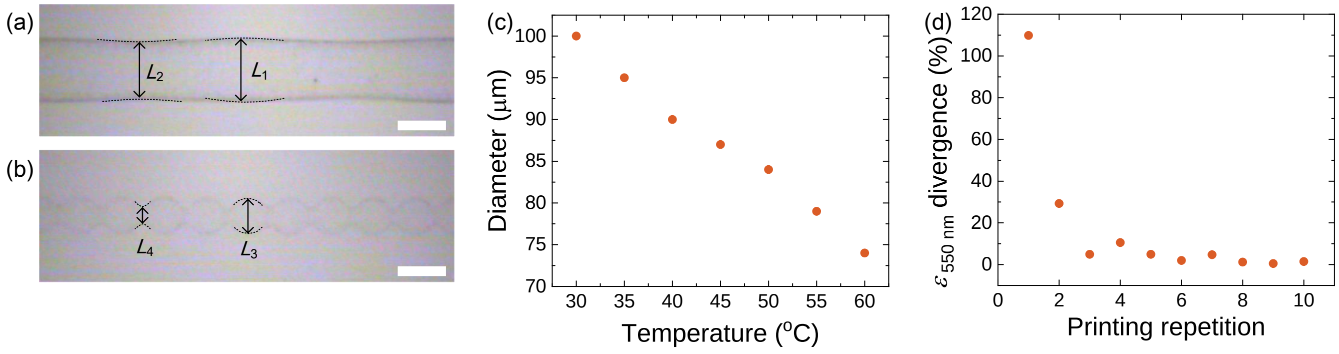

Supplementary Figure 9. Lines printed on untreated Si/SiO_2 with a droplet spacing of (a) 25 μm and (b) 85 μm, at 60°C, scale bar - 100 μm. The contrasts in (a,b) are enhanced for clarity; (c) Diameter of dried droplets under varied heating temperature; (d) Optical extinction divergence from the fitted extinction for 1-10 printing repetitions.

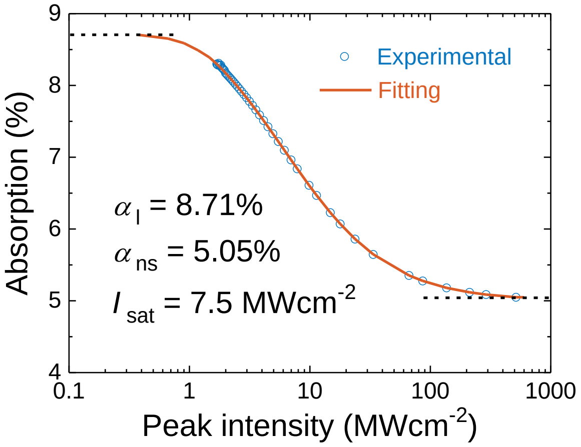

Supplementary Figure 10. Optical absorption profile of BP printed onto PET, obtained using a Z-scan experiment, the blue traces are the experimental data points while the red curve represents the fit.

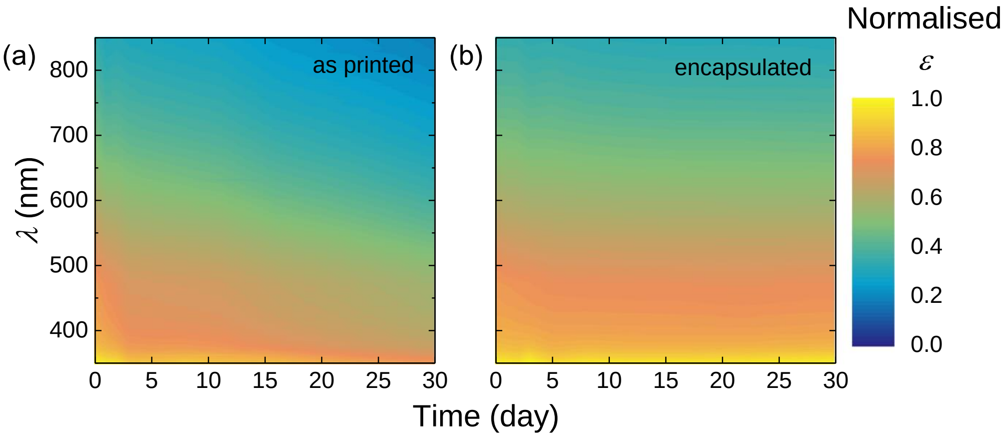

Supplementary Figure 11. Optical extinction spectrum for (a) as printed and (b) encapsulated printed BP under ambient conditions, the extinction is normalised to the initial extinction value at 350 nm at 0th day.

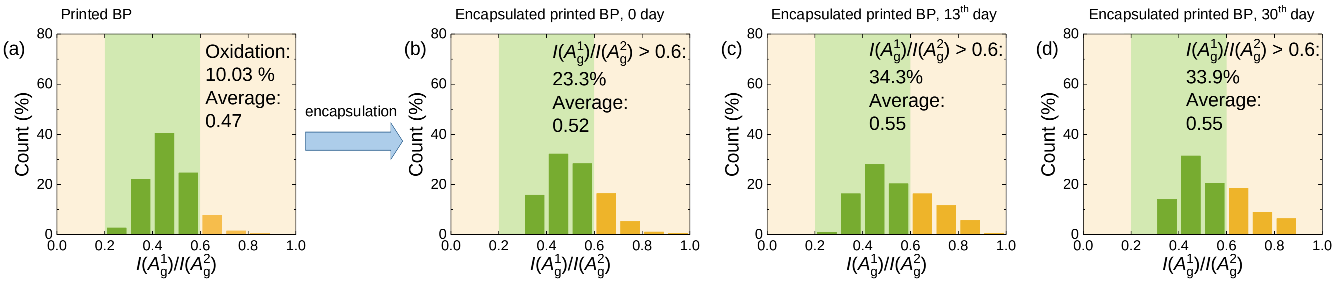

Supplementary Figure 12. $I(A_g^1)/I(A_g^2)$ histogram for (a) BP after printing, and (b) after encapsulation, (c) on 13th day and (d) on 30th day.

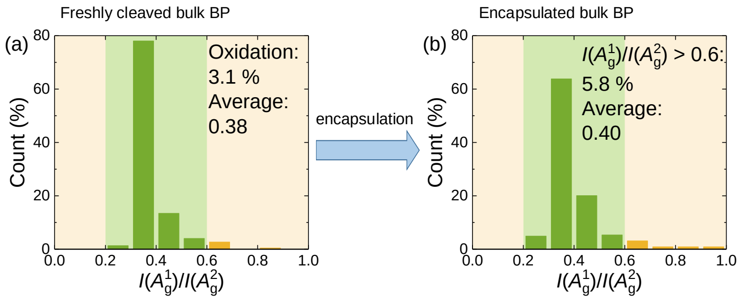

Supplementary Figure 13. $I(A_g^1)/I(A_g^2)$ histogram for (a) freshly cleaved bulk BP crystal and (b) immediately after parylene-C encapsulation. The increase in the ratio values indicate absolute $I(A_g^1)/I(A_g^2)$ values may not represent oxidation proportion of parylene-C coated BP samples.

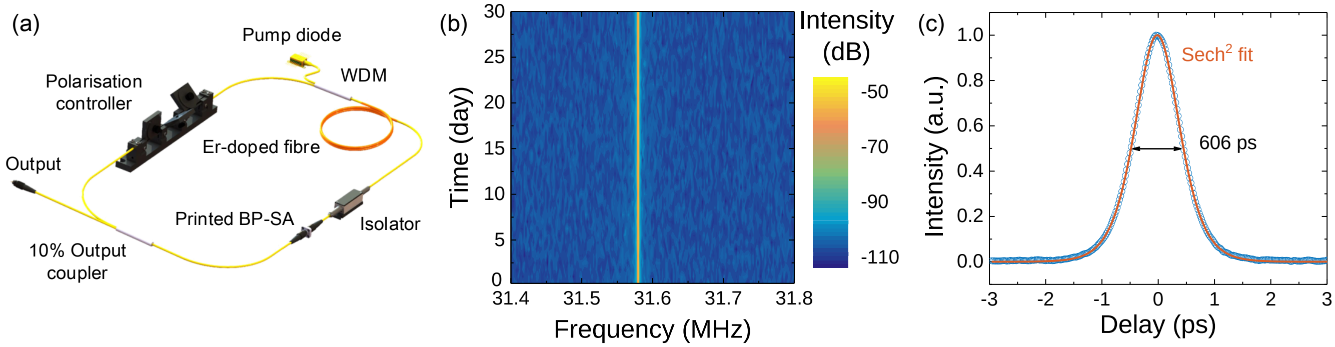

Supplementary Figure 14. Mode-locked ultrafast laser: (a) Schematic figure of a mode-locked erbium-doped ultrafast fibre laser set-up; (b) Stable radio frequency spectrum for 30 days; (c) Autocorrelation trace of output pulses, the blue traces for experimental data and the red curve for sech^2 fit.

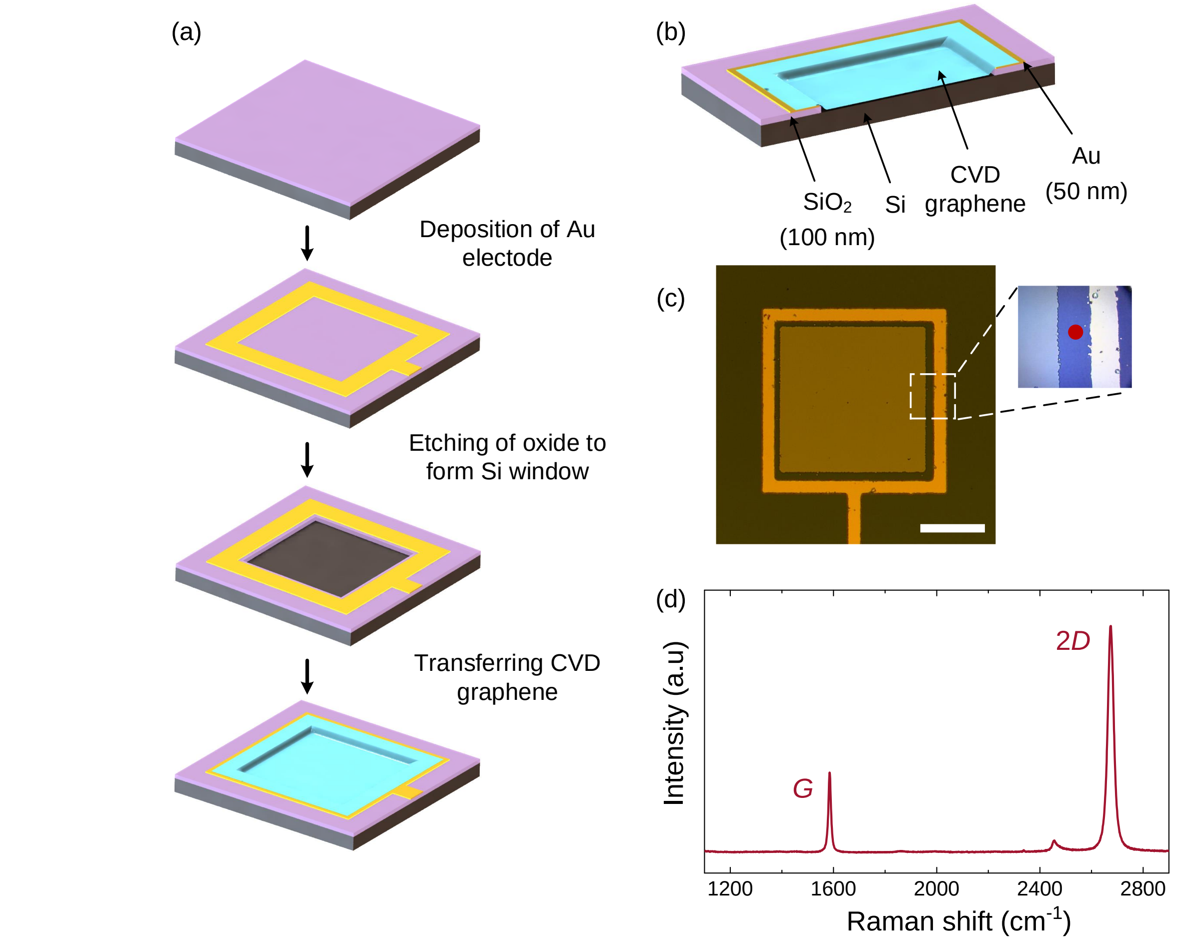

Supplementary Figure 15. Graphene/Si Schottky junction photodetector: (a) Device fabrication process; (b) Lateral height profile; (c) Top-view optical micrograph, scale bar - 200 μm. The red dot in the zoomed-in view indicates the position where Raman measurement is taken in (d); (d) Raman spectrum of the CVD graphene.

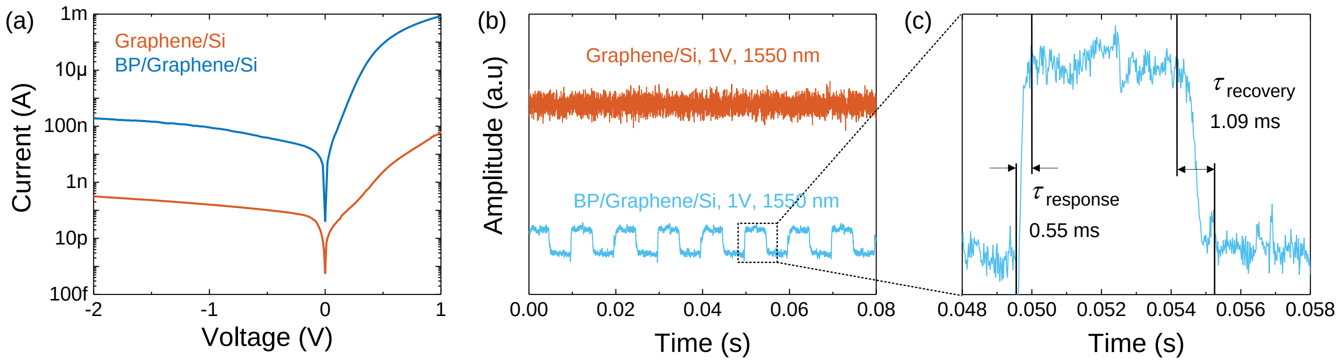

Supplementary Figure 16. (a) Dark current of Gr/Si and BP/Gr/Si; (b, c) Time response of Gr/Si and BP/Gr/Si.

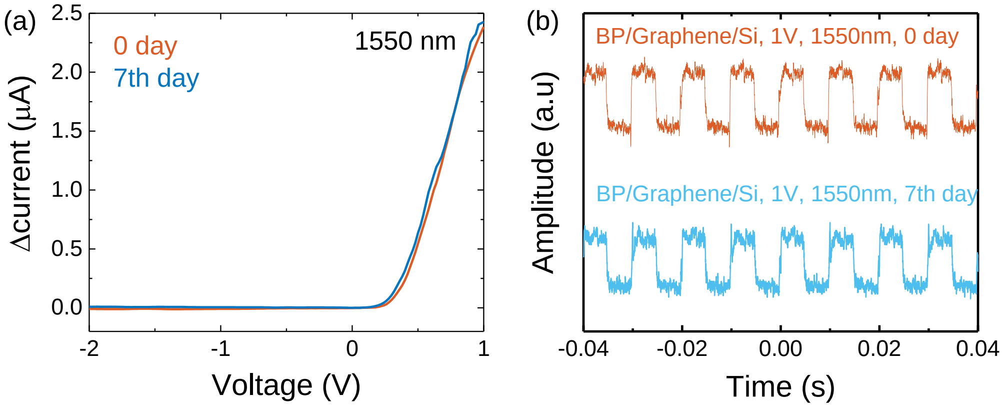

Supplementary Figure 17. BP/Gr/Si photodetection performance after 7 days exposed in open air: (a) photocurrent change; (b) time response.

Supplementary References

1. Curcio, J. A. & Petty, C. C. The near infrared absorption spectrum of liquid water. *J. Opt. Soc. Am.* **41**, 302-304 (1951).
2. Wozniak, B. & Dera, J. Light absorption by water molecules and inorganic substances dissolved in sea water. In *Light Absorpt. Sea Water*, 11-81 (Springer, New York, NY, 2007).
3. Hanlon, D. *et al.* Liquid exfoliation of solvent-stabilized few-layer black phosphorus for applications beyond electronics. *Nat. Commun.* **6**, 8563 (2015).
4. Bohren, C. E. & Wiley, D. R. H. *Absorption and scattering of light by small particles* (Wiley-VCH Verlag GmbH, Weinheim, Germany, 2007).
5. Friedlander, S. K. S. K. *Smoke, dust, and haze: Fundamentals of aerosol dynamics* (Oxford University Press, Oxford, UK, 2000).
6. Favron, A. *et al.* Photooxidation and quantum confinement effects in exfoliated black phosphorus. *Nat. Mater.* **14**, 826-832 (2015).
7. Ling, X. *et al.* Anisotropic electron-photon and electron-phonon interactions in black phosphorus. *Nano Lett.* **16**, 2260-2267 (2016).
8. Xia, F., Wang, H. & Jia, Y. Rediscovering black phosphorus as an anisotropic layered material for optoelectronics and electronics. *Nat. Commun.* **5**, 4458 (2014).
9. Yasaei, P. *et al.* High-quality black phosphorus atomic layers by liquid-phase exfoliation. *Adv. Mater.* **27**, 1887-1892 (2015).
10. Torrisi, F. *et al.* Inkjet-printed graphene electronics. *ACS Nano* **6**, 2992-3006 (2012).
11. Finn, D. J. *et al.* Inkjet deposition of liquid-exfoliated graphene and MoS_2 nanosheets for printed device applications. *J. Mater. Chem. C* **2**, 925-932 (2014).
12. Kang, J. *et al.* Solvent exfoliation of electronic-grade, two-dimensional black phosphorus. *ACS Nano* **9**, 3596-3604 (2015).
13. Garmire, E. Resonant optical nonlinearities in semiconductors. *IEEE J. Sel. Top. Quantum Electron.* **6**, 1094-1110 (2000).
14. Sheik-Bahae, M., Said, A., Wei, T.-H., Hagan, D. & Van Stryland, E. Sensitive measurement of optical nonlinearities using a single beam. *IEEE J. Quantum Electron.* **26**, 760-769 (1990).
15. Hasegawa, A. Transmission of stationary nonlinear optical pulses in dispersive dielectric fibers. I. Anomalous dispersion. *Appl. Phys. Lett.* **23**, 142 (1973).
16. Chen, Y. *et al.* Mechanically exfoliated black phosphorus as a new saturable absorber for both Q-switching and Mode-locking laser operation. *Opt. Express* **23**, 12823-12833 (2015).
17. Sotor, J., Sobon, G., Macherzynski, W., Paletko, P. & Abramski, K. M. Black phosphorus saturable absorber for ultrashort pulse generation. *Appl. Phys. Lett.* **107**, 051108 (2015).
18. Sotor, J. *et al.* Ultrafast thulium-doped fiber laser mode locked with black phosphorus. *Opt. Lett.* **40**, 3885-3888 (2015).
19. Li, D. *et al.* Polarization and thickness dependent absorption properties of black phosphorus: New saturable absorber for ultrafast pulse generation. *Sci. Rep.* **5**, 15899 (2015).
20. Qin, Z. *et al.* Mid-infrared mode-locked pulse generation with multilayer black phosphorus as saturable absorber. *Opt. Lett.* **41**, 56-59 (2016).
21. Hisyam, M. B., Rusdi, M. F. M., Latiff, A. A. & Harun, S. W. Generation of Mode-Locked Ytterbium doped fiber ring laser using few-layer black phosphorus as a saturable absorber. *IEEE J. Sel. Top. Quantum Electron.* **23**, 39-43 (2017).
22. Ismail, E. I., Kadir, N. A., Latiff, A. A., Ahmad, H. & Harun, S. W. Black phosphorus crystal as a saturable absorber for both a Q-switched and mode-locked erbium-doped fiber laser. *RSC Adv.* **6**, 72692-72697 (2016).
23. Lee, D., Park, K., Debnath, P. C., Kim, I. & Song, Y.-W. Thermal damage suppression of a black phosphorus saturable absorber for high-power operation of pulsed fiber lasers. *Nanotechnology* **27**, 365203 (2016).
24. Song, Y. *et al.* Vector soliton fiber laser passively mode locked by few layer black phosphorus-based optical saturable absorber. *Opt. Express* **24**, 25933-25942 (2016).
25. Mao, D. *et al.* Stable high-power saturable absorber based on polymer-black-phosphorus films. *Opt. Commun.* (2016).
26. Li, J. *et al.* Black phosphorus: a two-dimension saturable absorption material for mid-infrared Q-switched and mode-locked fiber lasers. *Sci. Rep.* **6**, 30361 (2016).

27. Luo, Z.-C. *et al.* Microfiber-based few-layer black phosphorus saturable absorber for ultra-fast fiber laser. *Opt. Express* **23**, 20030-20039 (2015).

28. Park, K. *et al.* Black phosphorus saturable absorber for ultrafast mode-locked pulse laser via evanescent field interaction. *Ann. Phys.* **527**, 770-776 (2015).

29. Yu, H., Zheng, X., Yin, K., Cheng, X. & Jiang, T. Thulium/holmium-doped fiber laser passively mode locked by black phosphorus nanoplatelets-based saturable absorber. *Appl. Opt.* **54**, 10290-10294 (2015).

30. Chen, Y. *et al.* Optically driven black phosphorus as a saturable absorber for mode-locked laser pulse generation. *Opt. Eng.* **55**, 081317 (2016).

31. Chen, Y., Chen, S., Liu, J., Gao, Y. & Zhang, W. Sub-300 femtosecond soliton tunable fiber laser with all-anomalous dispersion passively mode locked by black phosphorus. *Opt. Express* **24**, 13316-13324 (2016).

32. Ferrari, A. C. *et al.* Raman spectrum of graphene and graphene layers. *Phys. Rev. Lett.* **97**, 187401 (2006).

33. Engel, M., Steiner, M. & Avouris, P. Black phosphorus photodetector for multispectral, high-resolution imaging. *Nano Lett.* **14**, 6414-6417 (2014).

34. Buscema, M. *et al.* Fast and broadband photoresponse of few-layer black phosphorus field-effect transistors. *Nano Lett.* **14**, 3347-3352 (2014).

35. Guo, Q. *et al.* Black phosphorus mid-infrared photodetectors with high gain. *Nano Lett.* **16**, 4648-4655 (2016).

36. Viti, L. *et al.* Black phosphorus terahertz photodetectors. *Adv. Mater.* **27**, 5567-5572 (2015).

37. Youngblood, N., Chen, C., Koester, S. J. & Li, M. Waveguide-integrated black phosphorus photodetector with high responsivity and low dark current. *Nat. Photonics* **9**, 247-252 (2015).

38. Yuan, H. *et al.* Polarization-sensitive broadband photodetector using a black phosphorus vertical p-n junction. *Nat. Nanotechnol.* **10**, 707-713 (2015).

39. Deng, Y. *et al.* Black phosphorus-monolayer MoS_2 van der Waals heterojunction p-n diode. *ACS Nano* **8**, 8292-8299 (2014).

40. Ye, L., Li, H., Chen, Z. & Xu, J. Near-infrared photodetector based on MoS_2 /black phosphorus heterojunction. *ACS Photonics* **3**, 692-699 (2016).

41. McManus, D. *et al.* Water-based and biocompatible 2D crystal inks for all-inkjet-printed heterostructures. *Nat. Nanotechnol.* **12**, 343-350 (2017).

42. Choi, K.-H., Yoo, J., Lee, C. K. & Lee, S.-Y. All-inkjet-printed, solid-state flexible supercapacitors on paper. *Energy Environ. Sci.* **9**, 2812-2821 (2016).
